# CD5-Positive Primary Cutaneous Diffuse Large B-Cell Lymphoma-Leg Type

**DOI:** 10.1155/2019/3730915

**Published:** 2019-01-16

**Authors:** Alexandra Papoudou-Bai, Leonidas Marinos, Amalia Vassou, Eleni Kapsali, Panagiotis Kanavaros

**Affiliations:** ^1^Department of Pathology, Faculty of Medicine, School of Health Sciences, University of Ioannina, Ioannina, Greece; ^2^Department of Hematopathology, Evangelismos General Hospital, Athens, Greece; ^3^Department of Hematology, Faculty of Medicine, School of Health Sciences, University of Ioannina, Ioannina, Greece; ^4^Department of Anatomy-Histology-Embryology, Faculty of Medicine, School of Health Sciences, University of Ioannina, Ioannina, Greece

## Abstract

Most primary cutaneous B-cell lymphomas (PCBCL) are CD5 negative, and only a few cases were found to express CD5. We report the first well-documented CD5+ primary cutaneous diffuse large B-cell lymphoma-leg type (PCDLBCL-LT). A 71-year-old woman with a history of Multiple Sclerosis was admitted because of a nodule at the left thigh. Histological examination of the skin biopsy disclosed a diffuse dermal infiltration by large lymphoid cells. Immunohistochemistry revealed that these large cells were positive for CD5, CD20, CD79a, MUM1/IRF4, Bcl6, Bcl2, and cytoplasmic IgM/*λ*, whereas CD3, CD56, CD23, CD21, CD10, CD30, cyclin D1, CD68, lysozyme, myeloperoxidase, and CD34 were not detected. Thus, the diagnosis of a CD5+ PCDLBCL-LT was made. Despite treatment, the patient died 11 months after initial diagnosis.

## 1. Introduction

Primary cutaneous B-cell lymphomas (PCBCL) are the second most common form of primary cutaneous lymphomas (PCL) and account for approximately 25%-30% of all PCL [1, 2]. Low-grade PCBCL, namely, the primary cutaneous follicle center lymphoma (PCFCL) and the primary cutaneous marginal zone lymphoma (PCMZL), represent the vast majority of PCBCL and display an indolent clinical course and a very good prognosis [1–5]. In contrast to PCMZL and PCFCL, primary cutaneous diffuse large B-cell lymphoma-leg type (PCDLBCL-LT) have an unfavorable prognosis [1–5]. PCDLBCL-LT typically presents as nodules or tumors in one or two legs and approximately 10-15% of patients present with lesions outside the lower extremities [1–5]. The large neoplastic B-cells typically express CD20, CD79a, PAX5, MUM1/IRF4, Bcl2, and FOXP1 whereas BCL6 may be present or undetectable [1–5]. Interestingly, PCDLBCL-LT seems to be a “cutaneous counterpart” of activated B-cell-like nodal DLBCL with a similar cytogenetic and immunohistochemical profile [1–7]. Most PCDLBCL are CD5 negative and, to the best of our knowledge, only 5 cases of this tumor were found to express CD5 [1, 8–12]. We report the first well-documented CD5+ PCDLBCL-LT occurring in a 71-year-old woman with a history of Multiple Sclerosis.

## 2. Case Presentation

**Figure 1:**
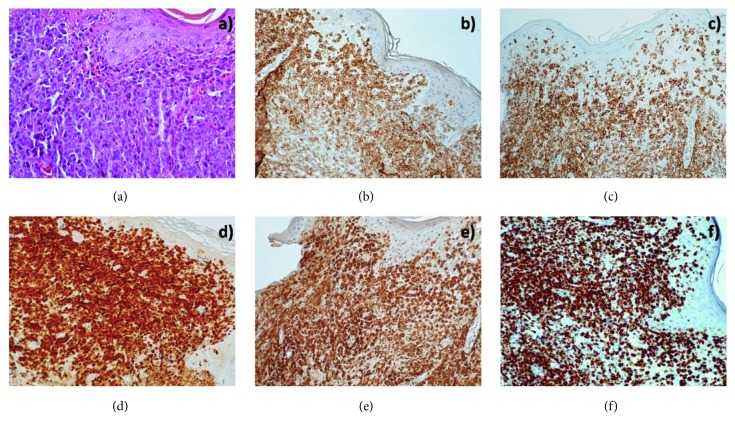
(a) Diffuse infiltration of the dermis by large lymphoid tumor cells with variable proportions of centroblasts and immunoblast-like cells (Hematoxylin-Eosin: magnification X400), (b–e) Positive immunostaining of large tumor cells for CD20, CD5, MUM1, and Bcl2, respectively (magnification X200) and (f) Ki67 immunostaining in approximately 90% of large tumor cells.

The patient was a 71-year-old woman with a history of Multiple Sclerosis during the past 12 years receiving dimethyl fumarate and baclofen. She also had hypertension and hyperlipidemia. She noticed a nodule at her left thigh, thought initially to be a mosquito bite. As the nodule grew bigger, a biopsy was performed. Histological examination demonstrated a diffuse dermal infiltration by large lymphoid cells (Figure 1(a)). Immunohistochemistry revealed that these large cells were positive for CD5, CD20, CD79a, MUM1/IRF4, Bcl6, Bcl2, and cytoplasmic IgM/*λ* whereas CD3, CD56, CD23, CD21, CD10, CD30, cyclin D1, CD68, lysozyme, myeloperoxidase, and CD34 were not detected (Figures 1(b)–1(e)). MYC immunopositivity was observed in 20% of tumor cells but our case was considered as MYC negative since the threshold for MYC immunohistochemical positivity in DLBCL is immunostaining of >40% of tumor cells [13]. Ki-67 immunostaining was detected in approximately 90% of large tumor cells (Figure 1(f)). Thus, the diagnosis of PCDLBCL-LT was made on the basis of clinical, histological, and immunohistochemical findings. The patient underwent Computed Tomography (CT) scans of thorax and abdomen and a bone marrow biopsy with no abnormal findings. She had four cycles of R-CHOP, with main side effect profound neutropenia. Ten months after the initial diagnosis she experienced right hemiplegia and entered the Neurology Department since worsening of Multiple Sclerosis was the primary diagnosis. A CT scan of the brain revealed lesions on basal ganglia and a biopsy was performed. On the basis of histological and immunohistochemical findings localization of DLBCL in Central Nervous system (CNS) was diagnosed. The patient was admitted to the Hematology Department and started treatment with methotrexate 3,5 mg/m^2^. After the first cycle, she experienced an episode of hematuria and urinary infection with Enterococcus faecalis. After completing the antibiotic treatment, a second methotrexate treatment was given followed by a third cycle on schedule. Ten days after the last treatment, the patient had epileptic seizures and was transferred to the Intensive Care Unit where she died five days later because of progressive disease.

## 3. Discussion

In the present report, the diagnosis of PCDLBCL-LT was established on the basis of clinical, histological, and immunohistochemical findings. The most relevant differential diagnosis of PCDLBCL-LT is PCFCL with diffuse growth pattern that is based on morphology (centroblast- and immunoblast-like cells for PCDLBCL-LT vs centrocyte-like cells for PCFCL) and the immunophenotype (PCDLBCL-LT display strong positivity for Bcl-2, MUM1/IRF4 and cytoplasmic IgM, whereas MUM1/IRF4 and cytoplasmic IgM are undetectable in PCFCL and most of PCFCL are negative for Bcl-2) [1, 3, 4]. Notably, it was emphasized that any PCDLBCL with positivity for both Bcl2 and MUM1/IRF4 should be designated as leg-type, regardless of anatomical localization [4]. A diagnostic challenge may also result from CD30 expression in large B-cell lymphoma resembling anaplastic large T-cell lymphoma, but expression of CD20 and absence of T-cell markers lead to the diagnosis [1].

Interestingly, our case is CD5+ whereas most PCDLBCL-LT are CD5 negative [1, 3, 4]. To the best of our knowledge, only five cases of CD5+ PCLBL have been reported so far [8–12]. However, it was not possible to establish the diagnosis of PCDLBCL-LT in these 5 previous cases since immunostaining results for both MUM1/IRF4 and Bcl2 were not reported in any case [8–12]. Therefore, our case is the first well-documented CD5+ PCDLBCL-LT.

Notably, all previously reported cases of CD5+ PCLBL responded well to chemotherapy [8–12]. However, our patient died 11 months after diagnosis although she was treated with R-CHOP and methotrexate. Thus, further studies with additional cases are required to determine if there are prognostic differences between CD5-positive and CD5-negative PCDLBCL-LT.

It is noteworthy that our patient had a history of Multiple Sclerosis and a previously reported patient had a history of Rheumatoid arthritis [9]. This observation raises the possibility that the pathogenesis of CD5+ PCLBL might be related to autoimmunity [14, 15]. In this regard, it is noteworthy that CD5 is a negative regulator of T-cell Receptor (TCR) and B-cell Receptor (BCR) signalling and using animal models evidence was provided that increased expression of CD5 on either T cells or B cells protects against autoimmunity as a consequence of an increase of the threshold needed for TCR- or a BCR-mediated activation following antigen recognition [14]. Of potential interest is also the finding that a significantly increased percentage of CD19+CD5+ cells has been detected by two-colour fluorescence analysis in patients with progressive Multiple Sclerosis [16]. Further studies are required to gain insight into the putative role of the autoimmunity in the pathogenesis of CD5+ PCLBL.

In conclusion, we present the first well-documented CD5-positive PCDLBCL-LT occurring in a 71-year-old woman with a history of Multiple Sclerosis. Despite treatment with R-CHOP and methotrexate, the patient died 11 months after diagnosis.
